# Measuring DHEA-S in saliva: time of day differences and positive correlations between two different types of collection methods

**DOI:** 10.1186/1756-0500-3-204

**Published:** 2010-07-20

**Authors:** Courtney A Whetzel, Laura C Klein

**Affiliations:** 1 Biobehavioral Health Department, 315 East Health and Human Development Building, The Pennsylvania State University, University Park, PA, USA

## Abstract

**Background:**

The anabolic steroid, dehydroepiandosterone sulfate (DHEA-S), is secreted from the adrenal cortex. It plays a significant role in the body as a precursor to sex steroids as well as a lesser known role in the hypothalamic pituitary adrenal axis (HPA) response to stress. DHEA-S can be measured reliably in saliva, making saliva collection a valuable tool for health research because it minimizes the need for invasive sampling procedures (e.g., blood draws). Typical saliva collection methods include the use of plain cotton swab collection devices (e.g., Salivette^®^) or passive drool. There has been some speculation that the plain saliva cotton collection device may interfere with determination of DHEA-S by enzyme immunoassay (EIA) bringing this saliva collection method into question. Because of the increasing popularity of salivary biomarker research, we sought to determine whether the cotton swab interferes with DHEA-S determination through EIA techniques.

**Findings:**

Fifty-six healthy young adult men and women aged 18-30 years came to the lab in the morning (0800 hrs; 14 men, 14 women) or late afternoon (1600 hrs; 14 men, 14 women) and provided saliva samples via cotton Salivette and passive drool. Passive drool collection was taken first to minimize particle cross contamination from the cotton swab. Samples were assayed for DHEA-S in duplicate using a commercially available kit (DSL, Inc., Webster, TX). DHEA-S levels collected via Salivette and passive drool were positively correlated (r = + 0.83, p < 0.05). Mean DHEA-S levels were not significantly different between collection methods. Salivary DHEA-S levels were significantly higher in males than in females, regardless of saliva collection method (p < 0.05), and morning DHEA-S values were higher than evening levels (p < 0.05).

**Conclusions:**

Results suggest that DHEA-S can be measured accurately using passive drool or cotton Salivette collection methods. Results also suggest that DHEA-S levels change across the day and that future studies need to take this time of day difference into account when measuring DHEA-S.

## Background

Dehydroepiandrosterone-sulfate (DHEA-S), the sulfated form of dehydroepiandrosterone (DHEA), is an important health biomarker used consistently in the measurement of allostatic load [1,2]. As a steroid, there has been interest in measuring DHEA-S in youth and aging studies [3-7]. Despite research interest in this biomarker, there is ambiguity regarding the best method to measure DHEA-S in saliva. For example, recent studies have incorporated salivary DHEA-S as an additional biomarker of hypothalamic-pituitary-adrenal axis (HPA) function [8-11], however their collection methods vary between passive drool and cotton salivette. This collection method difference is important because hormone recovery from saliva can vary by collection device [12-14]. Specifically, hormones such as sex steroids (e.g., testosterone and estrogen) and DHEA that are detectable in saliva must be collected through the passive drool method because they bind to the collection device and provide either falsely inflated or deflated values [15-17]. It is not known how these collection methods alter DHEA-S determination in saliva.

Not only is it important to validate DHEA-S in different types of saliva collection methods, but it also is important to understand how DHEA-S levels differ across sex and across the day. DHEA-S levels show a life-time rhythm with values high at birth, a sharp decline during the first year, a peak again in the mid 20-30s, and then a decline throughout the life span. In addition, males have higher levels of DHEA-S than do females [18]. Literature suggests that serum DHEA-S values are stable across the day due to a 10-12 hour renal clearance rate [19-21]. However, studies also suggest that levels of DHEA-S in saliva or serum do change across the day, with some reporting higher morning levels compared to evening and other reporting the opposite pattern [22-25]. The discrepancy between these studies could be the result of improved assay techniques, different sample populations or different sampling methods. Thus, it remains unclear whether DHEA-S values do change across this day. Therefore, we sought to determine whether DEHA-S could be reliably measured in saliva collected using two common saliva collection techniques: passive drool and cotton salivette. In addition, we examined salivary DHEA-S values in the morning and in the evening to determine DHEA-S changes across the day in a normal, health population of young adult men and women.

## Methods

### Participants

Fifty-six (28 females and 28 males) participants between the ages of 18-30 years (mean 21.77 ± 0.35 years) were recruited from The Pennsylvania State University Campus to take part in a 10-minute lab session.

### Experimental Procedure

Lab sessions were scheduled either between 0800-0900 hrs or 1600-1700 hrs. Each participant provided 2 saliva samples, one using the Salivette^® ^(Sarstedt, Newton, NC) with the cotton swab device in place to collect saliva (Salivette) and one using a piece of straw to spit into a Salivette^® ^ that had the cotton and plastic insert removed (passive drool). Following informed consent, participants were asked to think of their favorite food and, using a straw, spit into a collection tube for 2 minutes. After the passive drool collection, participants filled out a brief demographic survey that took approximately 5 minutes to complete. This brief time interlude allowed a wash out period between passive drool and Salivette saliva collection. Passive drool collection was taken first for all participants in order to minimize particle cross contamination from the cotton Salivette. Following the demographic survey, participants placed a cotton swab from a Salivette in their mouths without touching the cotton and rolled the swab across their tongue for 2 minutes (i.e., without chewing on the swab; unstimulated saliva collection).

All tubes were weighed before and after saliva collection to account for saliva volume (as per Harmon and colleagues) [26]. After both saliva samples were taken, collection tubes were placed in a -20 degree freezer and transferred to a -80 degree freezer within 24 hours for later assay. Participants were compensated $5.00 for their time. Study procedures were reviewed and approved by The Pennsylvania State University Institutional Review Board.

### Salivary DHEA-S Assessment

On the day of assay, samples were brought to room temperature and centrifuged for 5 minutes at 1500 x g to separate mucin from clear saliva. Salivary DHEA-S levels were evaluated in duplicate with sensitivity at 0.08 ng/mL by commercially available enzyme linked immunosorbent assay (EIA) kits (DSL, Webster, TX) in the core laboratory of The Pennsylvania State University General Clinical Research Center. Samples were balanced across assay plates so that each plate had the same number of morning and evening participants as well as male and female participants. Samples within each participant were kept together on a single assay plate.

### Statistical Analyses

**Figure 1:**
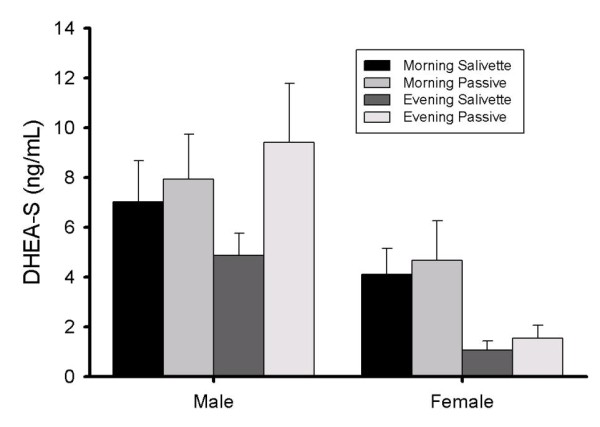
**Average DHEA-S (ng/mL) levels (± SEM) in male and female participants**.

Repeated-measures analysis of variance (RMANOVA) was used to determine differences in "type" (passive drool vs. Salivette) of saliva collection. Time of day (i.e., morning vs. evening participants) and sex were the independent variables, with levels of salivary DHEA-S collected by passive drool (time 1) and Salivette (time 2) as the dependent variables. Further analyses then were run to examine differences within each saliva collection type by performing separate 2-way ANOVAs (with time of day and sex as independent variables) on the passive drool collection and on the Salivette collection methods separately. Natural logarithmic transformations were applied to the data because they were skewed [27,28]; this transformation resulted in normal distribution of the data. Transformed data were used for analyses. However, raw data are reported in Figure 1 for clarity [27,28]. All tests were two-tailed and significance was determined at the alpha = 0.05 value.

## Results

DHEA-S levels were similar between passive drool and Salivette collection methods. Consistent with prior research, DHEA-S values among men were higher in both passive drool and Salivette saliva samples compared to women, regardless of time of day [F(1,50) = 10.56, p < 0.05] (see Figure 1) [18]. Further, DHEA-S values were higher for both collection techniques in the morning compared to the afternoon [F(1,50) = 9.27, p < 0.05] (see Figure 1). There were no statistically significant two-way interactions between saliva collection type and sex, saliva collection type and time of day, and no 3-way interactions among saliva collection type, sex or time of day collected. Pearson product-moment correlation confirmed a significant positive correlation between DHEA-S levels measured in passive drool and levels determined in saliva from the Salivette among all participants [r(55) = + 0.83, p < 0.001)]. Split by sex and time all four groups (i.e., morning male, evening male, morning female and evening female) passive drool and Salivette collected DHEA-S levels were also correlated within each group [r(14) = + 0.82; r(13) = + 0.83; r(13) = + 0.95; r(15) = + 0.95, respectively; p's < 0.001]. Saliva weights were measured on a subset of participants, resulting in the passive drool collection method yielding less saliva than did the Salivette collection device [1.42 g +.10 vs 2.45 g + .11 respectively, F(1,37) = 85.64, p < 0.05]. However, saliva weights were not correlated with DHEA-S values for either passive drool or Salivette [r(35) = -0.12, n.s.; r(37) = -0.12, respectively], and were not significant predictors of DHEA-S levels.

## Conclusions

Results suggest that both Salivette and passive drool collection result in similar DHEA-S levels in the morning and late afternoon and among men and women. These results are consistent with Shirtcliff and colleagues' [12] report that DHEA-S can be reliably measured in passive drool and cotton swab using radioimmunoassay (RIA) methods. Our study advances these findings in two important ways. First, we demonstrated reliable DHEA-S assessment in passive drool and cotton swab using enzyme immunolinked assay (ELISA) methods. Second, we used the standard cotton swab saliva collection procedure of rolling the swab over the tongue whereas Shirtcliff et al. [12] passed passive drool through a cotton swab. Together, our study and that of Shirtcliff et al. [12] suggest that DHEA-S can be reliably measured in saliva across these two collections and assay determination techniques.

Controversy still surrounds the type of collection device in regards to measuring hormones in saliva. Specifically, there are two issues: 1) low saliva volume may result in falsely low levels of hormones [26] and 2) blood contamination due to mucosal fissures of saliva samples could falsely inflate hormone values [12-14]. This study was designed to test the validity of DHEA-S in the Salivette compared to passive drool, however we also measured saliva volumes in order to record any differences in DHEA-S levels in lieu of the amount of saliva provided. Though blood contamination was not measured specifically in the current studies, unpublished studies in our lab have detected low levels of blood contamination in saliva samples collected in the field and that they do not appear to impact DHEA-S values.

Interestingly, results of this study also support data that report DHEA-S values may not be stable across the day as otherwise assumed in the literature (rhythm, [22,24,25]; no rhythm, [29-31]). Despite convincing literature reporting DHEA-S levels vary across the day, many studies have only measured DHEA-S at one time point [11,32-34]. Based on the limited prior studies and the present results, future studies need to take into consideration that DHEA-S levels may change across the day.

Salivette collection is a clean and easy method for collecting saliva (i.e., reduces pipette error due to mucin secretion), especially in field collection studies commonly designed for cortisol assessment [35]. If one is interested in measuring salivary sex steroids (i.e., estradiol, progesterone, testosterone), the Salivette is not recommended. However, several analytes can be measured reliably in salvia collected via Salivette such as salivary alpha-amylase, cortisol, DHEA-S and cotinine, the primary metabolite of nicotine found in cigarette smokers. The Salivette collection technique appears to be a clean and adequate collection method for determining DHEA-S.

## Abbreviations

HPA: Hypothalamic-pituitary-adrenal axis; DHEA-S: Dehyroepiandrosterone sulfate; EIA: enzyme immunosorbent assay; ANOVA: analysis of variance; RMANOVA: Repeated-measures analysis of variance

## Competing interests

The authors declare that they have no competing interests.

## Authors' contributions

LCK provided funding. CAW and LCK designed the experiment. CAW conducted the experiment and drafted the introduction and methods sections. CAW analyzed the data and drafted the results section. LCK made intellectual revisions to the entire manuscript. Both authors edited all sections of the manuscript and approved this version of the submitted manuscript.

## References

[B1] Seeman TE McEwen BS Singer BH Albert MS Rowe JW Increase in urinary cortisol excretion and memory declines. MacArthur studies of successful aging J Clin Endocrinol Metab 1997 82 8 2458 2465 10.1210/jc.82.8.2458 9253318

[B2] Seeman TE Singer BH Rowe JW Horwitz RI McEwen BS Price of adaptation-allostatic load and its health consequences. MacArthur studies of successful aging Arch Intern Med 1997 157 19 2259 2268 10.1001/archinte.157.19.2259 9343003

[B3] Almeida DM Piazza JR Dmitrieva ND Klein LC Frontiers in the use of biomarkers of health in research on stress and aging J Gerontol: Psych Sciences in press 10.1093/geronb/gbq049 PMC2920946 20647348

[B4] Di Luigi L Guidetti C Baldari MC Gallotta P Sgro P Perroni F Romanelli F Lenzi A Cortisol, dehydroepiandrosterone sulfate and dehydroepiandrosterone sulphate/cortisol ratio responses to physical stress in males are influenced by pubertal development J Endocrinol Invest 2006 29 796 804 17114910 10.1007/BF03347373

[B5] Huang YJ Chen MT Fang CL Lee WC Yang SC Kuo CH A possible link between exercise training adaptation and dehydroepiandrosterone-sulfate- an oldest-old female study Int J Med Sci 2006 3 4 141 147 17003845 10.7150/ijms.3.141 PMC1570619

[B6] Enomoto M Adachi H Fukami A Furuki K Satoh A Otsuka M Kumagae S Nanjo Y Shigetoh Y Imaizumi T Serum dehyroepiandrosterone sulfate levels predict longevity in men: 27-year follow-up study in a community-based cohort (Tanushimaru Study) J Am Geriatr Soc 2008 56 6 994 998 10.1111/j.1532-5415.2008.01692.x 18422949

[B7] Davis SR Shah SM McKenzie DP Kulkarni J Davison SL Bell RJ Dehydroepiandrosterone sulfate levels are associated with more favorable cognitive function in women J Clin Endocrinol Metab 2008 93 3 801 808 10.1210/jc.2007-2128 18073302

[B8] Assies J Visser I Nicolson NA Eggelte TA Wekking EM Huyser J Lieverse R Schene AH Elevated salivary dehydroepiandrosterone-sulfate but normal cortisol levels in medicated depressed patients: preliminary findings Psychiatry Res 2004 128 2 117 122 10.1016/j.psychres.2004.05.016 15488954

[B9] Taylor MK Sausen KP Potterat EG Mujica-Parodi LR Reis JP Markham AE Padilla GA Taylor DL Stressful military training: endocrine reactivity, performance, and psychological impact Aviat Space Environ Med 2007 78 12 1143 1149 10.3357/ASEM.2151.2007 18064919

[B10] Mommersteeg PM Heijnen CJ Kavelaars A van Doornen LJ The HPA-axis and immune function in burnout Prog Brain Res 2008 167 281 285 full_text 18037026 10.1016/S0079-6123(07)67024-1

[B11] Jeckel CM Lopes RP Berleze MC Luz C Feix L Argimon II Stein LM Bauer ME Neuroendocrine and immunological correlates of chronic stress in 'strictly healthy' populations Neuroimmunomodulation 2010 17 1 9 18 10.1159/000243080 19816052

[B12] Shirtcliff EA Granger DA Schwartz E Curran MJ Use of salivary biomarkers in biobehavioral research: cotton-based sample collection methods can interfere with salivary immunoassay results Psychoneuroendocrinology 2001 26 165 173 10.1016/S0306-4530(00)00042-1 11087962

[B13] Kivilighan KT Granger DA Schwartz EB Nelson V Curran M Shirtcliff EA Quantifying blood leakage into the oral mucosa and its effects on the measurement of cortisol, dehydroepiandrosterone, and testosterone in saliva Horm Behav 2004 46 39 46 10.1016/j.yhbeh.2004.01.006 15215040

[B14] Gallagher P Leitch MM Massey AE McAllister-Williams RH Young AH Assessing cortisol and dehydroepiandrosterone (DHEA) in saliva: effects of collection method J Psychopharmacol 2006 20 5 643 649 10.1177/0269881106060585 16401657

[B15] Lamey PJ Nolan A The recovery of human saliva using the Salivette system Eur J Clin Chem Clin Biochem 1994 32 9 727 728 7865631

[B16] Groschl M Rauh M Influence of commercial collection devices for saliva on the reliability of salivary steroids analysis Steroids 2006 71 13-14 1097 1100 10.1016/j.steroids.2006.09.007 17070563

[B17] Groschl M Kohler H Topf HG Rupprecht T Rauh M Evaluation of saliva collection devices for the analysis of steroids, peptides and therapeutic drugs J Pharm Biomed Anal 2008 47 3 478 486 10.1016/j.jpba.2008.01.033 18325706

[B18] Orentreich N Brind JL Rizer RL Vogelman JH Age changes and sex differences in serum dehydroepiandrosterone sulfate concentrations throughout adulthood J Clin Endocrinol Metab 1984 59 3 551 555 10.1210/jcem-59-3-551 6235241

[B19] Hornsby PJ Biosynthesis of DHEAS by the human adrenal cortex and its age-related decline Ann N Y Acad Sci 1995 774 29 46 10.1111/j.1749-6632.1995.tb17370.x 8597467

[B20] Baulieu EE Dehydroepiandrosterone (DHEA): a fountain of youth? J Clin Endocrinol Metab 1996 81 9 3147 3151 10.1210/jc.81.9.3147 8784058

[B21] Kroboth PD Salek FS Pittenger AL Fabian TJ Frye RF DHEA and DHEA-S: a review J Clin Pharmacol 1999 39 4 327 348 10.1177/00912709922007903 10197292

[B22] Patacchioli FR Monnazzi P Simeoni S De Filippis S Salvatori E Coloprisco G Martelletti P Salivary cortisol, dehydroepiandrosterone-sulphate (DHEA-S) and testosterone in women with chronic migraine J Headache Pain 2006 7 2 90 94 10.1007/s10194-006-0274-6 16575505 PMC3451699

[B23] Zhao ZY Xie Y Fu YR Li YY Bogdan A Touitou Y Circadian rhythm characteristics of serum cortisol and dehydroepiandrosterone sulfate in healthy Chinese men aged 30-60 years. A cross-sectional study Steroids 2003 68 2 133 138 10.1016/S0039-128X(02)00167-8 12606003

[B24] Del Ponte A Di Monte MG Graziani D Guagnano MT Menduni P Vitullo F Sensi S Changes in plasma DHEAS circadian rhythm in elderly men Prog Clin Biol Res 1990 341A 791 796 2145584

[B25] Nicolau GY Haus E Lakatua DJ Bogdan C Sackett-Lundeen L Popescu M Berg H Petrescu E Robu E Circadian and circannual variations of FSH, LH, testosterone dehydroepiandrosterone-sulfate (DHEA-S) and 17-hydroxy progesterone (17 OH-Prog) in elderly men and women Endocrinologie 1985 23 4 223 246 2935925

[B26] Harmon AG Hibel LC Rumyantseva O Granger DA Measuring salivary cortisol in studies of child development: Watch out- what goes in may not come out of saliva collection devices Dev Psychobiol 2007 49 5 495 500 10.1002/dev.20231 17577235

[B27] Klein LC Corwin EJ Ceballos RM Leptin, hunger, and body weight: Influence of gender, tobacco smoking, and smoking abstinence AddictBehav 2004 29 5 921 927 10.1016/j.addbeh.2004.02.023 15219336

[B28] Whetzel CA Corwin EJ Klein LC Disruption in Th1:Th2 immune response in young adult smokers Addict Behav 2007 32 1 1 8 10.1016/j.addbeh.2006.03.007 16644136

[B29] Goodyer IM Herbert J Altham PM Pearson J Secher SM Shiers HM Adrenal secretion during major depression in 8- to 16-year-olds, I. Altered diurnal rhythms in salivary cortisol and dehydroepiandrosterone (DHEA) at presentation Psychol Med 1996 26 2 245 256 10.1017/S0033291700034644 8685281

[B30] Hucklebridge F Hussain T Evans P Clow A The diurnal patterns of the adrenal steroids cortisol and dehydroepiandrosterone (DHEA) in relation to awakening Psychoneuroendocrinology 2005 30 1 51 57 10.1016/j.psyneuen.2004.04.007 15358442

[B31] Morgan CA Rasmusson A Pietrzak RH Coric V Southwick SM Relationships among plasma dehydroepiandrosterone and dehydroepiandrosterone sulfate, cortisol, symptoms of dissociation, and objective performance in humans exposed to underwater navigation stress Biol Psychiatry 2009 66 4 334 340 10.1016/j.biopsych.2009.04.004 19500775

[B32] Carlson LE Speca M Patel KD Goodey E Mindfulness-based stress reduction in relation to quality of life, mood, symptoms of stress and levels of cortisol, dehydroepiandrosterone sulfate (DHEAS) and melatonin in breast and prostate cancer outpatients Psychoneuroendocrinology 2004 29 4 448 474 10.1016/S0306-4530(03)00054-4 14749092

[B33] de Bruin VM Vieira MC Rocha MN Viana GS Cortisol and dehydroepiandosterone sulfate plasma levels and their relationship to aging, cognitive function, and dementia Brain Cogn 2002 50 2 316 323 10.1016/S0278-2626(02)00519-5 12464198

[B34] Cruess DG Antoni MH Kumar M Ironson G McCabe P Fernandez JB Fletcher M Schneiderman N Cognitive-behavioral stress management buffers decreases in dehydroepiandrosterone sulfate (DHEA-S) and increases in the cortisol/DHEA-S ratio and reduces mood disturbance and perceived stress among HIV-seropositive men Psychoneuroendocrinology 1999 24 5 537 549 10.1016/S0306-4530(99)00010-4 10378240

[B35] Strazdins L Meyerkort S Brent V D'Souza RM Broom DH Kyd JM Impact of saliva collection methods on sIgA and cortisol assays and acceptability to participants J Immunol Methods 2005 307 1-2 167 171 10.1016/j.jim.2005.09.010 16305798

